# Identification of common genetic modifiers of neurodegenerative diseases from an integrative analysis of diverse genetic screens in model organisms

**DOI:** 10.1186/1471-2164-13-71

**Published:** 2012-02-14

**Authors:** Xi Chen, Robert D Burgoyne

**Affiliations:** 1 Department of Cellular and Molecular Physiology, Physiological Laboratory, Institute of Translational Medicine, University of Liverpool, Crown St, Liverpool, L69 3BX, U.K

## Abstract

**Background:**

An array of experimental models have been developed in the small model organisms *C. elegans, S. cerevisiae *and *D. melanogaster *for the study of various neurodegenerative diseases including Alzheimer's disease, Parkinson's disease, and expanded polyglutamine diseases as exemplified by Huntington's disease (HD) and related ataxias. Genetic approaches to determine the nature of regulators of the disease phenotypes have ranged from small scale to essentially whole genome screens. The published data covers distinct models in all three organisms and one important question is the extent to which shared genetic factors can be uncovered that affect several or all disease models. Surprisingly it has appeared that there may be relatively little overlap and that many of the regulators may be organism or disease-specific. There is, however, a need for a fully integrated analysis of the available genetic data based on careful comparison of orthologues across the species to determine the real extent of overlap.

**Results:**

We carried out an integrated analysis using *C. elegans *as the baseline model organism since this is the most widely studied in this context. Combination of data from 28 published studies using small to large scale screens in all three small model organisms gave a total of 950 identifications of genetic regulators. Of these 624 were separate genes with orthologues in *C. elegans*. In addition, 34 of these genes, which all had human orthologues, were found to overlap across studies. Of the common genetic regulators some such as chaperones, ubiquitin-related enzymes (including the E3 ligase CHIP which directly links the two pathways) and histone deacetylases were involved in expected pathways whereas others such as the peroxisomal acyl CoA-oxidase suggest novel targets for neurodegenerative disease therapy

**Conclusions:**

We identified a significant number of overlapping regulators of neurodegenerative disease models. Since the diseases have, as an underlying feature, protein aggregation phenotypes it was not surprising that some of the overlapping genes encode proteins involved in protein folding and protein degradation. Interestingly, however, some of the overlapping genes encode proteins that have not previously featured in targeted studies of neurodegeneration and this information will form a useful resource to be exploited in further studies of potential drug-targets.

## Background

Despite major advances, debilitating neurodegenerative diseases including Alzheimer's disease (AD), Parkinson's disease (PD), and polyglutamine (polyQ) diseases as exemplified by Huntington's disease (HD) and related ataxias afflict millions worldwide and remains a significant and unresolved burden facing ageing populations. Many genetic factors including specific causative mutations have been identified but therapies for these debilitating and eventually fatal disorders are lacking. These disorders are associated with the unifying theme of accumulation of toxic, misfolded protein aggregates or inclusion bodies followed by progressive neuronal dysfunction, eventual neuronal loss and death. In many cases the mutations in disease-specific proteins that lead to protein aggregation have been indentified and there is growing evidence that the cellular protein quality control systems are an underlying common denominator of these diseases [1]. In different individuals, the susceptibility to disease-related mutations and the time of onset of age-related neurodegeneration differ significantly suggesting the importance of additional genetic factors or genetic variation [2]. Despite this, relatively few genetic factors shared between neurodegenerative diseases have been identified so far.

Knowledge of genetic regulators of neurodegeneration is important not only for an understanding of potential neuroprotective mechanisms but for the identification of potential new drug targets. The use of small model organisms with short generation times such as *C. elegans, S. cerevisiae *and *D. melanogaster*, has facilitated testing of hypotheses to illuminate a prospective cellular cause of protein-misfolding diseases like HD, PD, Amyotrophic Lateral Sclerosis (ALS) and AD or neuroprotective mechanisms against neurodegeneration [3,4]. Disease models in these organisms have also allowed screening of potential genetic modifiers of the late-onset cellular and behavioural phenotypes. Screening can be performed by molecular, genetic and chemical manipulations of gene function, i.e. using mutagenesis (deletion libraries, transposon based insertion), transgenic overexpression of exogenous human misfolding disease-related proteins, or RNA interference (RNAi)-mediated knockdown to determine the loss- or gain-of-function phenotypes. Previous studies have ranged from the examination of the effects of genetic manipulation of small number of targeted genes to whole genome screens. In addition, a wide range of disease models has been examined. For example in C. *elegans*, the most widely studied model organism in this context, multiple tissue-specific transgenic models manifesting pathological phenotypes that faithfully recapitulate many cellular and molecular pathologies of complex neurodegenerative disease processes have been used [5,6]. These models have been based on muscular or neuronal expression of aggregation-prone proteins such as mutant tau, superoxide dismutase (SOD1), α-synuclein, polyQ constructs, Huntingtin fragment and toxic amyloid beta 1-42 (Aβ1-42) [7] , and has allowed identification of modifiers and cellular processes of α-synuclein inclusion formation [8,9], polyQ [10] and mutant SOD1 aggregation [11], α-synuclein and polyQ-induced toxicity [12,13] and tau-induced pathology [14]. The model organism approach and the various disease models that have been studied have been extensively reviewed in recent years and will not be described further here [5,6,15-20].

It might be expected that common features should underlie the different pathways that lead to or protect from the phenotypes in diverse neurodegenerative diseases. One key question of interest, therefore, is the extent to which the model organism studies have identified common genetic regulators of neurodegenerative disease. While chaperone proteins involved in protein folding have emerged as common factors [1] it has appeared that relatively few overlapping genetic regulators have been identified from genetic screens using different models. For example the same group using a whole genome RNAi screen in *C. elegans *models of both polyQ and α-synuclein aggregation reported only a single gene overlap from a large number of hits identified [9,10]. Use of RNAi screens in *Drosophila *cell lines with similar huntingtin polyQ models identified 21 [21] and 126 [22] regulators respectively. Only three of these identified regulators overlapped between the two studies. In addition, only two regulators from the *Drosophila *cell line studies overlapped with those found in an assay for polyQ aggregation in human cells [23] and had opposite effects. In another study, regulation of toxicity due to expression of either α-synuclein or a huntingtin fragment in yeast was found to involve non-overlapping sets of genes [24]. One problem in comparing across all screens is the different disease models that have, for example, used toxicity in all or only in specific neurons or alternatively protein aggregate formation in either neurons or muscle as the disease phenotype [5,6,17]. An initial analysis of different screens based on the biological function of identified genes did indicate the role of common and differing functional classes of modifier genes involved in various cellular process including regulation of protein homeostasis, vesicular traffic and transcriptional control [17]. A significant problem in understanding the commonality of genetic regulators is the need for careful matching of protein orthologues across the three model species and this has not been carried out systematically.

We have set out to use data from published genetic screens in *C. elegans, S. cerevisiae *and *D. melanogaster *to generate an integrated data set of genetic regulators of neurodegeneration. In order to do this we have used *C. elegans *as the reference point since this organism is most applicable for large scale screens and has been very widely used for study of neurodegenerative mechanisms. For *C. elegans *and *D. melanogaster *we have focussed on screens at the whole organism level as these have the additional possibility of identifying non-cell autonomous factors. The aim of this study was to give an improved indication of shared genetic factors and to provide a resource for future studies on neurodegeneration and neuroprotection.

## Results and Discussion

**Figure 1:**
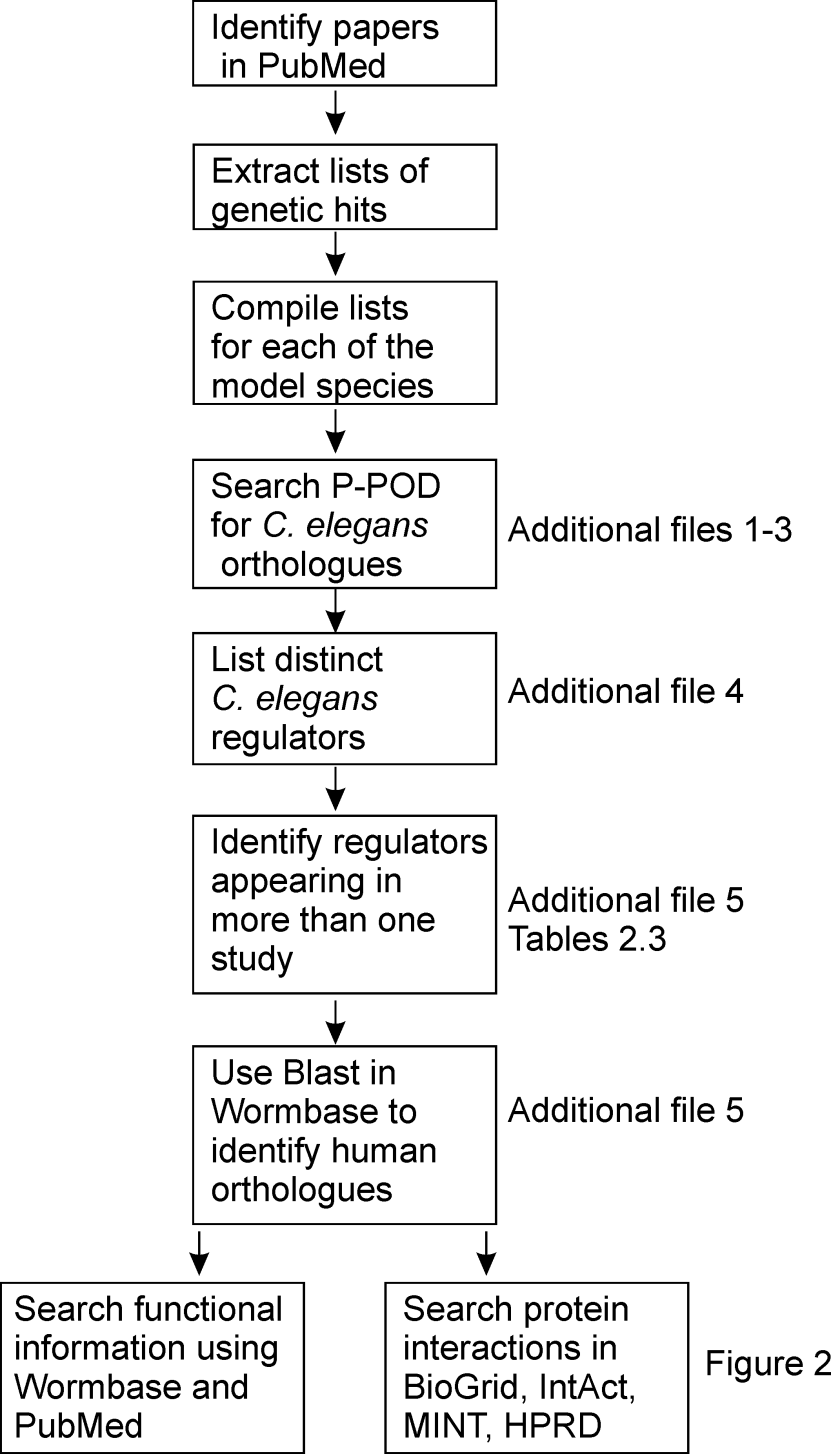
**Steps in the integrated analysis of genetic regulators identified from genetic approaches in model organisms**.

A series of papers were collated in which genetic approaches were used to identify regulators of neurodegenerative models in *S. cerevisiae *and diverse whole organism models in *C. elegans *and *D. melanogaster *(Figure 1). As well as medium and large scale screens we also included examples of targeted small scale genetic studies of candidate genes to allow inclusion of other regulators. There are additional candidate gene studies or screens on cells lines from *Drosophila *for example [21,22,25] that have not been included. Data from the published literature was first used to compile a list of genes identified as regulators in various neurodegeneration models in *C. elegans *(Additional file 1). For studies in *D. melanogaster *and *S. cerevisiae*, lists of the gene regulators was compiled and then the existence and identity of any *C. elegans *orthologues was examined for each genetic regulator using the Princeton Protein Orthology Database [26]. Confirmation of the existence of single or multiple potential orthologues generated lists of the *C. elegans *orthologues of genetic regulators of neurodegeneration from studies in *D. melanogaster *(Additional file 2) and *S. cerevisiae *(Additional file 3). From this analysis of a total of 950 identified genetic modifiers of neurodegeneration, 675 were found to have orthologues that could be identified in the *C. elegans *genome (Table 1). The genes that did not have identifiable orthologues in *C. elegans *are likely to be yeast- or fly-specific or simply not represented in the *C. elegans *genome.

**Table 1:** Genetic studies for the identification of regulators of neurodegeneration models in *C.elegans, S. cerevisiae *and *D. melanogaster*

Study Number	Primary model Organism	Disease model	Expressed construct	Screen	Number of Genetic modifiers	Orthologues in C. elegans	Reference
1	*C. elegans*	P	Htt-Q0, Q24, Q33, Q35, and Q40	Genome-wide RNAi	186	186	[10]
2	*C. elegans*	SOD	G85R SOD	Genome-wide RNAi	81	81	[11]
3	*C. elegans*	S	WT α-synuclein	Genome-wide RNAi	82	82	[9]
4	*C. elegans*	T	Tau P301L and V337M	Genome-wide RNAi	75	75	[14]
5	*C. elegans*	S	WT α-synuclein	Hypothesis-driven RNAi	20	20	[65]
6	*C. elegans*	P	Htt-Q150	Candidate genes	12	12	[37]
7	*C. elegans*	S	WT α-synuclein	Systematic RNAi screen	11	11	[68]
8	*C. elegans*	P	Htt-Q128	Candidate genes	4	4	[13]
9	*C. elegans*	Aβ	Aβ_1-42_	Candidate genes	2	2	[69]
10	*C. elegans*	T	Tau P301L and V337M	Forward genetic	2	2	[70]
11	*C. elegans*	T	Tau V337M	Candidate genes	2	2	[71]
12	*C. elegans*	P	Htt-Q32, Q40, Q56, Q79	Candidate genes	2	2	[72]
13	*C. elegans*	P	Htt-Q2 and Q150	Candidate genes	1	1	[12]
14	*S. cerevisiae*	P and S	Htt-Q20, Htt-Q53, A53T α-synuclein	Genome-wide genetic	138	41	[24]
15	*S. cerevisiae*	S	α-synuclein	Selected genetic screen	77	20	[73]
16	*S. cerevisiae*	Aβ	Aβ1-42	Genome-wide genetic	40	11	[60]
17	*S. cerevisiae*	P	Htt-Q103	Genome-wide loss-of-function suppressor	30	5	[50]
18	*S. cerevisiae*	S	WT α-synuclein	Genome-wide overexpression	22	7	[58]
19	*S. cerevisiae*	S	WT α-synuclein	Candidate genes	5	1	[59]
20	*D. melanogaster*	T	Tau V337M	Large-scale genetic	30	14	[74]
21	*D. melanogaster*	P	Htt-Q128	protein interaction and selected genetic screen	32	34	[75]
22	*D. melanogaster*	P	*SCA1*82Q and Htt-Q128	Selective genetic modifier screen	24	20	[34]
23	*D. melanogaster*	T	Tau V337M	Large-scale genetic	24	10	[76]
24	*D. melanogaster*	P	Htt-Q127	Selective genetic modifier screen	10	2	[77]
25	*D. melanogaster*	P	SCA3trQ78	Genome-wide screen	18	17	[63]
26	*D. melanogaster*	P	SCA1 30Q and 82Q	Large-scale genetic	17	11	[78]
27	*D. melanogaster*	P	Htt-Q127	Genome-wide screen	2	1	[79]
28	*D. melanogaster*	S	A30P and A53T α-synuclein	Candidate gene	1	1	[80]
Total					950	675	

The number of regulators of neurodegeneration that were identified in each study is shown along with the number of these genes with orthologues in C elegans. The disease models include organisms expressing forms of β-amyloid (AB), polyQ expansions (P), wild type or mutant α-synuclein (S), mutant superoxide dismutase (SOD), or mutant tau (T).

**Figure 2:**
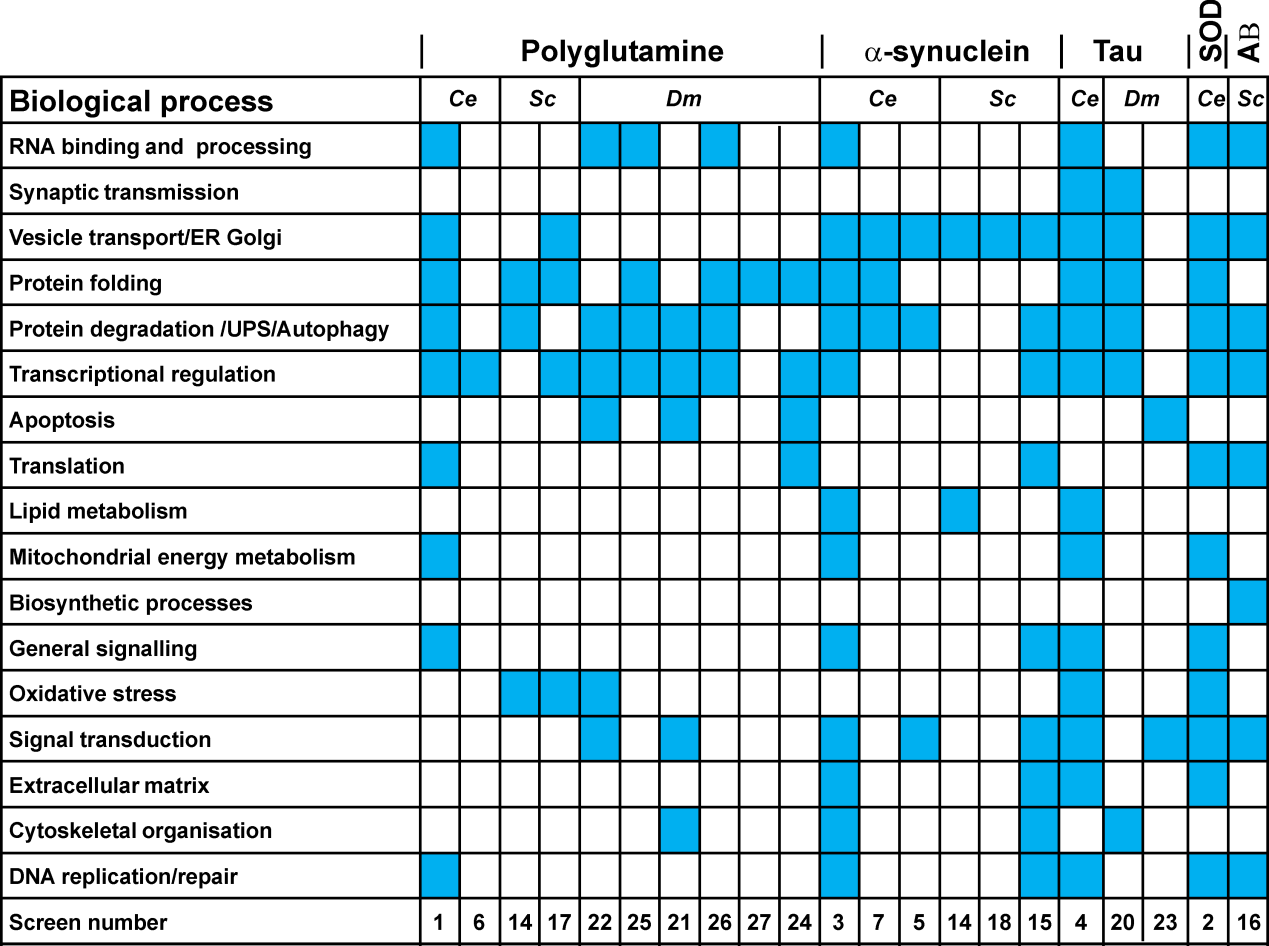
**Functional profiling of genetic modifiers identified in diverse screens**. Comparative analysis of modifiers identified in worm (*Ce*) models of polyQ, tau, SOD and α-synuclein aggregation, yeast (*Sc*) models of misfolded α-synuclein and Htt toxicity and fly (*Dm*) models of misfolded tau and polyQ toxicity from diverse screens reveals that the identified modifier genes function in a wide variety of biological processes as defined in the original studies. The blue filled box indicates that one or more genes in this category were identified. The number of the study indicated refers to the numbering in Table 1.

From the combined lists, a total of 624 distinct genes encoding genetic regulators were identified (Additional file 4). An initial analysis of the genetic modifiers based on cellular function was carried out. As described previously from this type of analysis [17] it could be seen that the genes covered a wide range of cellular functions covering 17 different classes of biological function (Figure 2). There was, however, a concentration of genes in certain functional classes. Genes involved in protein folding (e.g. heat shock proteins), protein degradation and autophagy were discovered across multiple disease models. Genes involved in transcriptional regulation were identified across all the polyQ and tau-disease models. It was noteworthy that the α-synuclein disease models produced a particular concentration of regulators functional in vesicular transport (e.g. *rab-1*) although these did appear, albeit less frequently, in studies on other disease models.

Of more interest was the potential identification of specific genes with overlapping modifying roles in different disease models and model organisms. Within the set of distinct *C. elegans *orthologues we found 34 that had been indentified in more than one study. These are shown in Table 2 and in an expanded version with additional information in Additional file 5. Significantly, all of these genes have human orthologues (Additional file 5). The overlapping regulators fell into several different functional classes (Table 3) based on their classification in the original studies.

**Table 2:** Overlapping genetic regulators of neurodegeneration with orthologues in *C.elegans*

Sequence Name	Gene name	Description	Organism	Disease model	Expression in neurons
Y53C10A.12	*hsf-1*	Heat-shock transcription factor	worm	Aβ, P, S, SOD, T	yes
F26D10.3	*hsp-1*	HSP70 protein family	fly, worm	P, T, S	yes
F54D5.8	*dnj-13*	Molecular chaperone (DnaJ)	fly	P, T	?
K01C8.10	*cct-4*	Chaperone containing TCP-1	worm	P, SOD	yes
C07G2.3	*cct-5*	Chaperone containing TCP-1	worm	P, SOD	?
T09B4.10	*chn-1*	C-term of HSP-70 interacting protein (E3 ubiquitin ligase)	fly, worm	P, T	yes
Y94H6A.6	*ubc-8*	Ubiquitin conjugating enzyme	fly, yeast	P, S	?
M7.1	*let-70*	E2 Ubiquitin conjugating enzyme	fly	P	yes
C06A1.1	*cdc-48.1*	AAA ATPase, functions as a ubiquitin-related chaperone	worm	P	Yes
F52D10.3, M117.2	*ftt-2, par-5*	14-3-3 family	fly	P	yes
C39F7.4	*rab-1*	Small Ras-like GTPase Rab1	fly, yeast, worm	P, S	yes
B0361.10	*ykt-6*	SNARE protein YKT6	yeast	S	?
C54H2.5	*sft-4*	SURF-4 related to cargo transport protein ERV29	worm, yeast	S	yes
Y54E2A.12	*tbc-20*	Predicted GTPase activator protein	yeast	S	?
R11A8.4	*sir-2.1*	NAD-dependent histone deacetylase	fly, worm	P, S	Yes
C53A5.3	*hda-1*	Histone deacetylase 1	fly, worm	P, S	No?
F02E9.4	*sin-3*	SIN3 family of histone deacetylase subunits	fly	P, S	yes
F59F4.1	-	Acyl-CoA oxidase	fly, yeast, worm	P, S, T	?
ZK256.1a	*pmr-1*	Golgi P-type ATPase Ca2+ -pump	yeast	S	yes
W08D2.5,	*catp-6*	Predicted lysosomal P-type ATPase	worm, yeast	S	?
Y43F4B.4	*npp-18*	Nuclear pore complex protein	fly	P	yes
F49E10.5	*ctbp-1*	Transcriptional co-repressor homolog	fly	P	?
K08F8.6	*let-19*	Transcriptional cofactor	fly	P	yes
T17E9.1	*kin-18*	Serine-threonine protein kinase	fly, worm	P, T	yes
H18N23.2	-	Protein phosphatase, regulatory subunit PPP1R3C/D	fly, worm	P, SOD	?
T14F9.1	*vha-15 (phi-52)*	Vacuolar ATPase subunit H	worm	P, S	Yes
T21E12.4	*dhc-1*	Dynein heavy chain	fly, worm	P	Yes
Y113G7B.18	*mdt-17*	MeDiaTor	worm	S, SOD	?
Y116A8C.35	*uaf-2*	U2AF splicing factor	worm	P, S	Yes
F56C11.1	*bli-3*	Homologue of dual oxidase	worm	P, SOD	No
C32E8.10c	*unc-11*	Clathrin-adaptor protein AP180	worm, yeast	Aβ, P	Yes
JC8.10b	*unc-26*	Synaptojanin	worm, yeast	Aβ, P	Yes
ZK742.1a	*xpo-1*	Exportin-1	Fly, yeast	Aβ, P	?
C05C8.7	*phi-49*	Mannose-6-phosphate isomerase	worm	P, SOD	No

The genes were included if they were identified in more than one genetic screen. The disease models included organisms expressing forms of β-amyloid (AB), polyQ expansions (P), wild type or mutant α-synuclein (S), mutant superoxide dismutase (SOD), or mutant tau (T). Under expression in neurons a question mark indicates that no data are available in Wormbase.

**Table 3:** Biological processes associated with the identified overlapping genetic regulators of neurodegeneration.

Biological process	Genes
Heat shock transcription factor	*hsf-1*
Molecular chaperones	*hsp-1, dnj-13, cct-4, cct-5*
Ubiquitin related	*chn-1, ubc-8, let-70, cdc-48.1*
Histone deacetylases	*sir2.1, had-1, sin-3*
Signalling/chaperone	*fft-2/par-5*
Transcriptional cofactor/regulator	*ctbp-1, let-19, mdt-17, uaf-2*
Nucleopore/nuclear export	*npp-18, xpo-1*
Vesicular transport	*rab-1, sft-4, tbc-20, ykt-6, dhc-1*
Endocytosis	*unc-11, unc-26*
Transport ATPase	*catp-6, pmr-1, vha-15*
Protein phosphorylation	*kin-18, H18N13.2*
Other cellular processes	*bli-3, phi-49, F59F4.1*

A recent study has extended the identification of suppressors of polyQ aggregation in *C. elegans *[10] by examining whether knock down of their human orthologues would suppress aggregation of mutant huntingtin in a human cell line. Of the 177 human orthologues, 26 inhibited aggregation in the HK293 cells supporting the idea that genetic regulators identified in *C. elegans *would have a conserved function relevant for a human model [23]. Three of the human suppressors correspond to the overlapping regulators in Table 2 (*hsp-1, cct-4 and cct-5*) and a fourth was an additional subunit of TCP-1 (*cct-2*).

Many but not all of the overlapping genes in Table 2 are known to be expressed in adult neurons in *C. elegans *where they could, therefore, have a physiological role in regulating neurodegeneration. This is clearly an important consideration as some of the worm disease models are based on aggregate formation in muscle rather than neuronal cells. It should be noted that the data available in WormBase on the cellular expression of worm proteins is variable and so the question of neuronal expression is uncertain for some of the regulators. Two genes, *bli-3 *and *phi-49 *have, however, non-neuronal and restricted cell type expression. This may suggest that they may be unlikely to be physiological regulators of neurodegeneration in the worm but alternatively they could, for example, affect release of extracellular factors that act on neurons.

**Figure 3:**
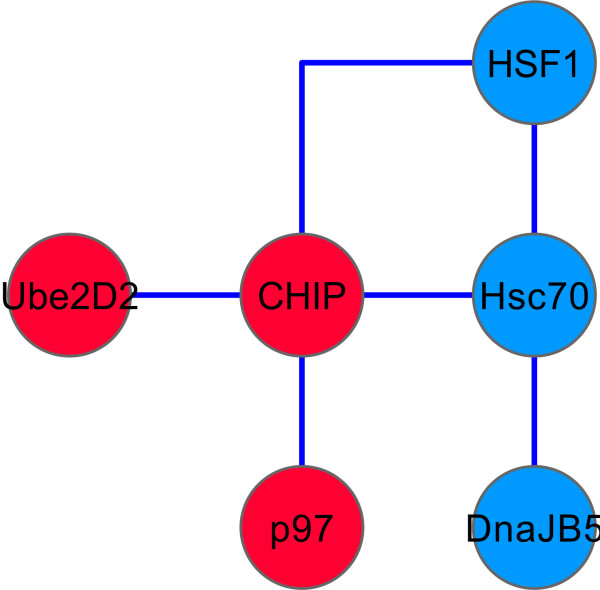
**The diagram shows known binary protein-protein interactions between the molecular chaperone and ubiquitin-related overlapping regulators in Table 2**. The interactions are included if they were identified between orthologues of the proteins from any species and are shown labelled with the names of the human orthologues with those involved in protein folding (chaperones) in blue and those in ubiquitin-related pathways in red. The human orthologues and their corresponding *C. elegans *orthologues are as follows: HSF1, *hsf-1*; Hsc70, *hsp-1*; DnaJB5*, dnj-13*; CHIP, *chn-1*; p97, *cdc-48.1*; Ube2D2, *let-70*.

The overlapping gene set included regulators identified in more than one study but only using the same or similar type of disease model in a single species (Table 2). Others, however, had been identified in multiple models and/or species. Amongst the latter were, unsurprisingly, members of families with functions related to protein folding such as *hsp-1, hsf-1, dnj-13, cct-4*, and *cct-5 *which have key roles in proteostasis [27]. In addition, three genes encoding enzymes involved in ubiquitination, *chn-1, ubc-8, and let*-70 were identified in more than one study. The mammalian orthologues, CHIP and Ube2D2 respectively, of *chn-1 *(an E3 ligase) and *let-70 *(E2 conjugating enzyme) are known to interact directly [28,29] as are CHIP and p97 (*cdc-48.1*) [30]. Interestingly, CHIP also provides a further link between ubiquitination and protein folding pathways based on its known interactions with and ability to ubiquitinate Hsc70 and HSF1 (Figure 3). These data would put CHIP at a key point in two pathways that modify neurodegeneration. The idea that CHIP is a key player in the regulation of neurodegeneration is reinforced by the evidence that it has been shown to ubiquitinate α-synuclein [31] and ataxin-1 [32] and to stimulate the ubiquitin ligase activity of the PD gene product Parkin [33]. In addition, CHIP has a neuroprotective role in the neurotoxicity caused by over-expression of ataxin-1 in a fly model [32]. Taken together with the screens included here [14,34] in which CHIP orthologues were found to modify neurodegeneration models including those based on tau-induced pathology, polyQ-related disorders and α-synuclein over-expression, it appears that CHIP could play a key protective role in multiple neurodegenerative diseases.

Another significant functional grouping in the shared modifiers is the three histone deacetylases, *sir-2.1, hda-1 *and *sin-3 *which were identified in both polyQ and α-synuclein disease models in flies and worms. The mammalian orthologues of *hda-1 *(human HDAC1) and *sin3 *(human SIN3B) interact directly and function as part of a protein complex to repress gene transcription [35,36]. A selective study on histone deacetylases in *C. elegans *showed opposing effects of different deacetylases but loss of either *hda-1 *or *sir-2.1 *exacerbated neurodegeneration due to polyQ toxicity [37]. Overexpression of *sir-2.1 *had been suggested to increase longevity in *C. elegans *[38] leading to widespread study of the potential anti-aging effects of the related sirtuins in many species including a possible role in the effects of calorie-restriction on life span in *C. elegans *[39] and other species [40]. It might be thought that its neuroprotective effect could be related to its general effect on ageing. Recent work has, however, largely eliminated a role for *sir-2.1 *in increasing lifespan following the removal of an additional unrelated mutation in the worm strain studied. In contrast, a neuroprotective effect on a polyQ model of neurodegeneration remained associated with *sir-2.1 *overexpression [41]. A neuroprotective role of calorie restriction in *C. elegans *has also been shown to be mediated by *sir-2.1 *[42]. SIRT1, the mammalian orthologues of *sir-2.1 *and other histone deacetylases have been well established to regulate neurodegeneration and have been suggested as potential drug targets [43,44]. The use of histone deacetylases inhibitors is currently being examined for treatment of neurodegenerative diseases but there are concerns about the potential detrimental effects of these inhibitors [43,45].

The epsilon isoform of the 14-3-3 proteins (CG31196) was identified in screens in the fly as a regulator of polyQ-mediated neurotoxicity. This fly protein has two worm orthologues (*ftt-2 *and *par-5*). One of these, *ftt-2 *has also been shown to be neuroprotective when over-expressed in a worm model of α-synuclein-mediated neurotoxicity [46]. It has been shown that 14-3-3 proteins may increase lifespan and can interact with *sir-2.1 *[47,48]. Recent work [49] has suggested that the neuroprotective role of 14-3-3 proteins in mammalian cells may be due to inhibition of the apoptotic factor Bax [49].

A screen of genetic regulators of toxicity due to a mutant huntingtin fragment in *S. cerevisiae *identified Bna4 (kynurenine 3-monooxygenase) as the most potent suppressor [50]. Orthologues of Bna4 exist in *D. melanogaster *(CG1555) and in *C. elegans *(R07B7.5) but they were not identified in any of the screens in these organisms in Table 1. Nevertheless, recent studies have identified neuroprotective effects of inhibition or loss of kynurenine 3-monooxygenase and the kynurenine pathway in general in a Huntington's disease model in *D. melanogaster *and both Huntington's and Alzheimer's disease models in mice [51,52] suggesting a similar role in different organisms. Significantly, the study in *D. melanogaster *[52] also showed that loss of function in the tryptophan 2,3-dioxygenase gene (vermillion) the first enzyme in the kynurenine pathway was neuroprotective. The *C. elegans *orthologue of tryptophan 2,3-dioxygenase (C28H8.11) was one of the suppressors of α-synuclein inclusion formation identified in a genome wide screen in *C. elegans *[9]. These two enzymes can be regarded, therefore, as general regulators of neurodegeneration across species and diseases.

Recent work has implicated defects in autophagy as a contributor to neurodegeneration and the process of autophagy as a key protective mechanism in preventing neurodegeneration [53-56]. Within the list of regulatory genes identified none encoded direct components of the autophagy machinery. Interestingly, however, the list of overlapping genes included *sir-2.1 *orthologues of which are involved in signalling pathways that control autophagy and thereby lifespan [53,57]. In addition, *rab-1 *was discovered in genetic screens as a regulator of α-synuclein-mediated protein aggregation in a yeast model and is also effective in neuroprotection in worms and flies [58,59]. The orthologues of *rab-1 *(specifically Rab1a in mammals) were recently shown to rescue an autophagy defect due to α-synuclein overexpression in mammalian cells and in *Drosophila *implicating the Rab1a isoform in autophagosome formation [54].

A genome-wide screen for genes that modify toxicity of Aβ1-42 in *S. cerevisiae *has identified 23 suppressor and 17 enhancer genes [60]. Of these 12 have human and 11 *C. elegans *orthologues. Three of the conserved yeast suppressors (*YAP1802, INP52 *and *SLA1*) have known functions in endocytosis. In addition, the human orthologues (*PICALM, SYNJ1 *and *SH3KBP1*) have been found from genome-wide association studies to be risk factors themselves (*PICALM*) or alternatively (*SYNJ1 *and *SH3KBP1*) to interact with known risk factors for Alzheimer's disease [61,62]. Examination of the effect the *C. elegans *orthologues (*unc-11, unc-26 *and *Y44E3A.4*) in a worm Aβ_1-42 _model confirmed that the endocytic genes had protective roles in this species [60]. Interestingly, a protective role for *unc-11 *and *unc-26 *has previously been identified in a *C. elegans *huntingtin polyQ disease model [13]. Overall these studies suggest an important role for clathrin-mediated endocytosis in regulation of toxicity in different disease models.

The other overlapping modifiers in Table 2 do not fit obviously into related functional classes but some of these genes may be generally significant. One that is noteworthy is the Acyl-CoA oxidase that was identified in all three model species in different disease models. The single orthologue in yeast, pox1, was identified as a regulator of α-synuclein toxicity in yeast [24]. The fly orthologue FBgn0027572 (CG5009) was identified in a genome wide screen for regulators of polyQ ataxin-3-mediated neurodegeneration in the eye where its over-expression suppressed the phenotype [63]. In addition, CG5009 also suppressed tau-mediated toxicity. Search of the Princeton Protein Orthology Database identifies seven orthologues of the yeast and fly genes in *C. elegans *(C48B4.1, F08A8.2, F08A8.3, F08A8.4, F08A8.1, F59F4.1, F25C8.1) and an additional orthologue has been postulated (F58F9.7) [64]. It is possible that all of the predicted worm acyl CoA oxidases could have overlapping functions. Of these orthologues, however, only the worm F59F4.1 gene was identified as a regulator of α-synuclein aggregate formation in a large scale RNAi screen where its knock down enhanced aggregation [65] suggesting that this particular orthologue has a non-redundant role. Interestingly in the fly model CG5009 was functionally linked to protein folding mechanisms based on an examination of effects of its over-expression on defects due to expression of a dominant negative form of Hsp70 [63]. The effect of over-expression of CG5009 was to enhance the defect, an effect that was also seen with the Hsp70 co-chaperone DnaJ-1. A single orthologue (ACOX1) is present in mammals where it is localised to peroxisomes and functions in β-oxidation of fatty acids. The potential importance of peroxisomes in general and more specifically acyl-CoA oxidase in neuroprotection is highlighted by the Zellweger class of peroxisomal biogenesis disorders in which there are severe neurological abnormalities. Mutations in ACOX1 (OMIM number 609751) are liked to clinical conditions which include a range of neurological problems. The function of acyl-CoA oxidase in fatty acid metabolism and its widespread tissue distribution results, however, in a range of clinical symptoms in conditions of reduced enzyme activity [66]. Acyl CoA oxidase has not previously been given serious consideration as regulator of neurodegeneration in specifically-targeted studies.

## Conclusions

An integrated analysis of data available from genetic screens of regulators of neurodegeneration in various disease models in yeast, fly and worms has identified 34 shared regulators. This is a higher number than was expected from previous studies. There are some additional candidate gene studies and screens on cell lines that were not included in this analysis and so the number could potentially be increased by incorporation of these. Several of the shared regulators identified here are members of classes of genes encoding proteins involved in protein folding and/or ubiquitin-dependent pathways. These form a group of 6 directly interacting proteins with the E3 ligase CHIP linking the two functional pathways. Other shared regulators that have received previous attention in targeted studies on neuroprotection included histone deacetylases and 14-3-3 proteins. Interestingly, other shared regulators emerged which have not previously been considered as key regulators of neurodegeneration including acyl-CoA oxidase. The role in neuroprotection of acyl-CoA oxidase, in particular, clearly warrants further study.

## Methods

### Literature Mining

Studies using genetic approaches to study genetic regulators of neurodegenerative disease models in *C. elegans, S. cerevisiae *and *D. melanogaster *were identified using key word searches in PubMed http://www.ncbi.nlm.nih.gov/sites/entrez). The identified published literature was manually curated to compile a collection of experimentally delineated genetic modifiers of protein aggregation, misfolding and neurodegeneration in *C. elegans, S. cerevisiae *and *D. melanogaster*. Files containing full lists of modifiers in the online supplemental materials of the papers were converted and imported into Microsoft Excel.

### Orthologue search

Individual worm orthologues of yeast and fly modifier genes were identified by searching the Princeton Protein Orthology Database (P-POD version 4) (http://ortholog.princeton.edu/findorthofamily.html) [26] and use of OrthoMCL [67] to determine the appropriate orthologues. These identifications were further checked and refined by consulting WormBase (http://www.wormbase.org/) using the "best Blast score", Saccharomyces Genome Database (SGD) (http://www.yeastgenome.org) and FlyBase (http://flybase.org) and use of BLAST searches (http://blast.ncbi.nlm.nih.gov/Blast.cgi). Information on the list of modifiers was further refined by conducting search queries in the bioinformatic interfaces at WormBase [WormBase Web site, available at http://www.wormbase.org, release version WS224, April, 2011], Biomart [http://www.biomart.org/], and GExplore 1.1 [http://genome.sfu.ca/gexplore/].

### Analysis of protein-protein interactions

Known protein-protein interactions amongst orthologues of the overlapping genetic regulators was identified by manual searches using the protein interaction databases BioGrid, IntAct, MINT, the Human Protein Reference Database and by literature searches on PubMed

## Abbreviations

AD: Alzheimer's disease; ALS: Amyotrophic Lateral Sclerosis; HD: Huntington's disease: PD: Parkinson's disease; polyQ: polyglutamine; SOD: superoxide dismutase.

## Competing interests

The authors declare that they have no competing interests.

## Authors' contributions

XC carried out bioinformatic analyses and interpretation of the data and contributed to the preparation of the manuscript. RDB carried out bioinformatic analyses, interpretation of the data and prepared the final version of the manuscript. Both authors read and approved the final version of the manuscript

## Supplementary Material

Additional File 1 **Table listing regulators of neurodegeneration from genetic studies in *C. elegans***. Click here for file

Additional File 2 **Table listing regulators of neurodegeneration from genetic studies in *D. melanogaster***. Click here for file

Additional File 3 **Table listing regulators of neurodegeneration from genetic studies in *S. cerevisiae***. Click here for file

Additional File 4 **Complied list of all distinct genetic regulators from studies in all three species with orthologues in *C. elegans***. Click here for file

Additional File 5 **Table listing shared genetic regulators found in more than one study of the three model species**. Click here for file
